# Uncoupling Erythropoiesis from Cardiorenal Effects: SGLT2 Inhibition in Non-Diabetic Heart Failure

**DOI:** 10.3390/medicina62050993

**Published:** 2026-05-19

**Authors:** Dan Claudiu Măgureanu, Ioana Corina Bocsan, Raluca Maria Pop, Maria Adriana Neag, Angela Cozma, Anca Dana Buzoianu

**Affiliations:** 1 Pharmacology, Toxicology and Clinical Pharmacology, Department of Morphofunctional Sciences, “Iuliu Hațieganu” University of Medicine and Pharmacy, Victor Babes, No 8, 400012 Cluj-Napoca, Romania; dan.clau.magureanu@elearn.umfcluj.ro (D.C.M.); raluca.pop@umfcluj.ro (R.M.P.); maria.neag@umfcluj.ro (M.A.N.); abuzoianu@umfcluj.ro (A.D.B.); 2 Department of Internal Medicine, “Iuliu Hațieganu” University of Medicine and Pharmacy, 400012 Cluj-Napoca, Romania; angelacozma@yahoo.com

**Keywords:** SGLT2 inhibitors, erythropoiesis, non-diabetic heart failure, hemoglobin, cardiorenal physiology

## Abstract

*Background and Objectives:* SGLT2 inhibitors increase hemoglobin and hematocrit in multiple clinical settings, an effect increasingly attributed to stimulation of erythropoiesis rather than hemoconcentration. However, most mechanistic evidence derives from diabetic populations, leaving uncertainty as to whether this response depends on diabetes-related metabolic changes. To evaluate whether dapagliflozin stimulates erythropoiesis in non-diabetic patients with heart failure and to determine whether hematologic changes correlate with renal, cardiac, inflammatory, hepatic, or iron-related parameters. *Materials and Methods:* In this retrospective observational study, each of 68 non-diabetic heart failure patients served as their own control. Hematologic, renal, cardiac, inflammatory, hepatic, and iron parameters were assessed at three time points: one year prior to dapagliflozin initiation, at baseline, and one year after initiation of therapy. Changes were analyzed using paired tests and correlation analyses. *Results:* Hemoglobin, hematocrit, and red blood cell count were significantly lower at the baseline compared with values recorded one year before dapagliflozin initiation and increased significantly during the year following treatment (all *p* < 0.001), while mean corpuscular indices remained stable. Serum iron decreased before treatment and increased significantly after dapagliflozin initiation (*p* < 0.05 vs. baseline); however, changes in serum iron did not correlate significantly with changes in hemoglobin after treatment. Inflammatory markers showed a modest reduction in C-reactive protein after treatment, while composite inflammatory indices remained largely stable. Liver enzymes showed no significant longitudinal changes. Correlation analyses demonstrated no association between changes in hemoglobin and changes in eGFR (ρ = 0.202, *p* = 0.098) or NT-proBNP (ρ = −0.003, *p* = 0.981) after treatment. Hematologic variables remained strongly intercorrelated, whereas cross-system correlations were minimal, indicating that erythropoietic stimulation occurred largely independently of renal or cardiac functional trajectories. *Conclusions:* Dapagliflozin robustly stimulates erythropoiesis in non-diabetic patients with heart failure, independent of improvements in kidney or cardiac function. Although serum iron levels improved after treatment, the absence of a direct correlation with hemoglobin suggests that iron mobilization may act as a permissive rather than a primary driver of erythropoietic response. These findings support the concept that erythropoiesis represents a diabetes-independent pharmacologic action of SGLT2 inhibitors and may involve renal, hepatic, inflammatory, and iron-regulatory pathways beyond those described in diabetic physiology. Dedicated mechanistic studies in non-diabetic populations are warranted.

## 1. Introduction

Sodium–glucose cotransporter 2 (SGLT2) inhibitors have rapidly evolved from antihyperglycemic agents to foundational therapies for cardiovascular and renal disease [1,2,3,4,5]. Beyond their established benefits in reducing hospitalization for heart failure and slowing kidney disease progression, SGLT2 inhibitors consistently increase hemoglobin and hematocrit across multiple clinical settings [6,7,8,9]. Initially interpreted as a consequence of hemoconcentration due to natriuresis [10,11,12], this effect is now widely believed to reflect enhanced erythropoiesis, supported by transient rises in erythropoietin (EPO) [13] and improved iron mobilization [14] reported in several trials involving patients with type 2 diabetes mellitus (T2DM) or chronic kidney disease (CKD).

Recent mechanistic analyses, particularly those by Packer and Vallon, propose that SGLT2 inhibition alleviates metabolic stress in proximal tubular cells, improves renal cortical oxygenation, and modulates hypoxia-inducible factor (HIF) signaling, thereby promoting renal and possibly hepatic EPO synthesis [15,16]. Importantly, most mechanistic data supporting these concepts derive from diabetic populations, in which hyperglycemia and elevated tubular glucose load fundamentally shape renal energetics and oxygen balance. It remains unclear whether these diabetes-specific factors are essential for the erythropoietic response or whether SGLT2 inhibitors activate additional, diabetes-independent pathways.

Recent trials in heart failure populations, including those with a substantial proportion of non-diabetic individuals, suggest that erythropoiesis may be a class effect across diverse clinical phenotypes [17,18]. However, studies specifically designed to examine erythropoietic responses in exclusively non-diabetic patients are limited, and the extent to which hematologic changes are independent of concurrent cardiorenal improvement remains insufficiently explored.

The present study addresses this knowledge gap by examining erythropoietic, renal, and cardiac trajectories in non-diabetic patients with heart failure treated with dapagliflozin. Using a self-controlled, before-and-after design spanning two consecutive years, we assessed whether increases in hemoglobin and related hematologic indices occur in the absence of corresponding improvements in renal or cardiac function. By focusing on a non-diabetic cohort, this study seeks to clarify whether the erythropoietic effects of SGLT2 inhibitors occur independently of diabetes and to offer new insights into the physiological processes behind this effect.

## 2. Materials and Methods

This was a retrospective, observational, self-controlled before-and-after study conducted at a single tertiary hospital. The study was strictly non-interventional. Dapagliflozin was prescribed as part of routine clinical care in accordance with contemporary heart failure guidelines, in patients considered clinically eligible by the treating cardiologist, typically in the context of persistent symptoms, functional deterioration, or optimization of guideline-directed medical therapy. Treatment decisions were made independently of the present analysis, and no therapeutic interventions were influenced by study participation. Each patient served as their own control, and the observation period extended over two years: one year before and one year after the initiation of dapagliflozin therapy. The study aimed to evaluate the changes and correlations between erythropoiesis, renal, and cardiac parameters in non-diabetic patients with heart failure.

Patients were retrospectively and consecutively identified among those who initiated dapagliflozin therapy between January 2022 and December 2024. Eligible patients had a confirmed diagnosis of heart failure, absence of diabetes mellitus, and continuous cardiology follow-up for at least 12 months before and 12 months after dapagliflozin initiation, with complete clinical and laboratory data available at all three predefined time points. Patients were excluded if they had a diagnosis of diabetes mellitus, experienced modifications of guideline-directed medical therapy during the observation period, initiated additional treatments known to significantly influence erythropoiesis, or had incomplete follow-up data. In addition, patients with acute inflammatory or infectious conditions, active malignancy, or severe hepatic disease were excluded in order to minimize potential confounding factors affecting erythropoiesis and iron metabolism.

Clinical, laboratory, and treatment-related data were extracted from electronic health records and systematically entered into a structured database. The following variables were analyzed: hematological parameters—hemoglobin, hematocrit, mean corpuscular volume (MCV), mean corpuscular hemoglobin (MCH), mean corpuscular hemoglobin concentration (MCHC), and red blood cell count (RBC); liver parameters—alanine aminotransferase (ALT) and aspartate aminotransferase (AST), inflammatory parameters—C-reactive protein (CRP), erythrocyte sedimentation rate (ESR), neutrophil-to-lymphocyte ratio (NLR), platelet-to-lymphocyte ratio (PLR), and systemic immune-inflammation index (SII); renal parameters—serum creatinine, serum urea, and estimated glomerular filtration rate (eGFR); serum iron; and cardiac parameters—clinical stage of heart failure, left ventricle ejection fraction (LVEF), and NT-proBNP concentrations. Measurements were obtained at three predefined time points: one year before initiation of dapagliflozin (−12 months), at baseline (initiation of dapagliflozin), and one year after initiation (+12 months).

A total of 68 patients with a confirmed diagnosis of heart failure were included. All patients were non-diabetic and were followed longitudinally within the same cardiology department. At baseline, all participants were receiving stable, guideline-directed medical therapy for heart failure, including angiotensin-converting enzyme inhibitors or angiotensin receptor blockers, beta-blockers, mineralocorticoid receptor antagonists, and diuretics, without any planned modifications. The sole therapeutic intervention during the observation period was the initiation of dapagliflozin at a dose of 10 mg once daily, which defined the baseline time point (T0). No other cardiovascular or non-cardiovascular treatments were initiated, discontinued, or up-titrated during the study interval. Data were collected for the year preceding dapagliflozin initiation (−12 months) and for one year following initiation (+12 months). Patients with incomplete follow-up, missing laboratory data, or any additional treatment changes during the observation period were excluded to ensure that dapagliflozin represented the only pharmacologic modification.

Statistical analysis was performed using intra-subject comparisons. Continuous variables were tested for normality using the Shapiro–Wilk test. Normally distributed variables were expressed as mean ± standard deviation and compared using paired Student’s t-tests. Non-normally distributed data were presented as median with interquartile range and analyzed using the Wilcoxon signed-rank test. Correlations between changes in erythropoietic, renal, and cardiac variables were assessed using Pearson’s or Spearman’s correlation coefficients, depending on distribution. A two-sided *p*-value <0.05 was considered statistically significant. All statistical analyses were performed using SPSS version 29 (IBM Corp., Armonk, NY, USA).

## 3. Results

### 3.1. Baseline Characteristics

Baseline demographic, clinical, and treatment characteristics of the study population are summarized in Table 1. A total of 68 non-diabetic patients with heart failure were included, with a mean age of 70.7 ± 10.9 years and a predominance of female patients (58.8%). The cohort was largely overweight, with a mean body mass index of 29.1 ± 6.2 kg/m^2^.

Cardiometabolic comorbidities were highly prevalent. Hypertension affected nearly 90% of patients, most frequently at moderate to severe stages, while dyslipidemia and non-alcoholic steatotic liver disease were present in approximately one third of the cohort. Chronic kidney disease was documented in 39.7% of patients, predominantly at moderate stages, and atrial fibrillation was present in over half of the population (52.9%).

At baseline, patients were receiving stable guideline-directed medical therapy for heart failure. The majority were treated with angiotensin-converting enzyme inhibitors or angiotensin receptor antagonists (85.3%), beta-blockers (91.2%), mineralocorticoid receptor antagonists (70.6%), and loop diuretics (61.8%). Sacubitril/valsartan was prescribed in one quarter of patients, and statin therapy was used in over 70%.

### 3.2. Cardiovascular Parameters

As summarized in Table 2, functional and cardiac parameters exhibited a biphasic pattern, with deterioration during the year preceding dapagliflozin initiation and subsequent improvement after one year of treatment. Functional status, assessed by NYHA class, worsened significantly in the year prior to treatment and improved significantly after dapagliflozin initiation (both *p* < 0.001), while no significant difference was observed between the pre-treatment and post-treatment periods (*p* = 0.095). Left ventricular systolic function followed a similar trajectory, with a significant decline before baseline and a significant improvement after dapagliflozin therapy (both *p* < 0.001), whereas LVEF values one year after treatment did not differ significantly from those recorded one year before baseline (*p* = 0.51). NT-proBNP levels mirrored this pattern, showing a marked increase prior to treatment initiation and a significant reduction after dapagliflozin therapy (both *p* < 0.001), with no significant difference between pre-treatment and post-treatment values (*p* = 0.144).

### 3.3. Renal Parameters

As shown in Table 3, renal parameters also followed a biphasic trajectory, characterized by deterioration during the year preceding dapagliflozin initiation and subsequent improvement after one year of therapy. Serum urea remained stable prior to treatment (*p* = 0.767) but decreased significantly after dapagliflozin initiation compared with both baseline (*p* = 0.003) and the pre-treatment period (*p* = 0.012). Serum creatinine increased significantly before treatment initiation (*p* < 0.001) and declined significantly after one year of dapagliflozin therapy (*p* = 0.002), reaching values not significantly different from those observed one year before baseline (*p* = 0.268). A similar pattern was observed for estimated glomerular filtration rate, which declined significantly prior to treatment (*p* < 0.001) and improved after dapagliflozin initiation compared with both baseline (*p* = 0.039) and the pre-treatment period (*p* = 0.029).

### 3.4. Liver Parameters

Liver enzymes remained stable across the study period (Table 4). Aspartate aminotransferase (AST) showed no significant variation between one year before dapagliflozin initiation, baseline, and one year after treatment. Alanine aminotransferase (ALT) demonstrated a mild downward trend over time, with slightly lower median values after dapagliflozin initiation and at one-year follow-up compared with the pre-treatment period; however, these changes did not reach statistical significance.

### 3.5. Inflammatory Status

As summarized in Table 5, inflammatory parameters remained largely stable over the study period, with limited changes observed after dapagliflozin initiation. C-reactive protein (CRP) showed a modest but statistically significant reduction after one year of dapagliflozin therapy compared with baseline (*p* = 0.035). However, CRP values one year after therapy did not differ significantly from those observed one year before dapagliflozin initiation. Erythrocyte sedimentation rate (ESR) did not change significantly across the three evaluated time points. No statistically significant differences were observed between one year before and baseline, baseline and one year after treatment, or between one year before and one year after dapagliflozin initiation. Similarly, neutrophil-to-lymphocyte ratio (NLR) remained stable throughout the study period, with no significant differences detected between any of the time points assessed. Platelet-to-lymphocyte ratio (PLR) also showed no significant variation over time. The systemic immune–inflammation index (SII) did not demonstrate significant changes during follow-up. Median SII values were comparable at one year before, at dapagliflozin initiation, and after one year of treatment, with no statistically significant differences between periods.

### 3.6. Hematological Parameters

As shown in Table 6, hematologic parameters exhibited a clear biphasic evolution across the study period. Red blood cell count, hemoglobin, and hematocrit declined significantly during the year preceding dapagliflozin initiation and increased significantly after one year of therapy. At baseline, median hemoglobin was 13.0 g/dL, hematocrit 40.4%, and red blood cell count 4.43 × 10^6^/µL, all significantly lower than one year before treatment (all *p* ≤ 0.005). After one year of dapagliflozin therapy, hemoglobin increased to 14.3 g/dL, hematocrit to 43.65%, and red blood cell count to 4.75 × 10^6^/µL, exceeding both baseline and pre-treatment values (all *p* ≤ 0.001). In contrast, erythrocyte indices reflecting red cell morphology remained largely unchanged. Mean corpuscular volume and mean corpuscular hemoglobin showed no significant variation over time, while mean corpuscular hemoglobin concentration demonstrated a modest but statistically significant reduction after dapagliflozin therapy compared with both baseline (*p* = 0.040) and the pre-treatment period (*p* = 0.001).

### 3.7. Serum Iron Status

As summarized in Table 7, serum iron levels also displayed a biphasic pattern across the study period. Serum iron decreased significantly from one year before dapagliflozin initiation to baseline, indicating a decline in iron availability prior to treatment (*p* = 0.007). Following one year of dapagliflozin therapy, serum iron levels increased significantly compared with baseline (*p* = 0.04), returning to values comparable to those observed one year before treatment initiation (*p* = 0.988).

### 3.8. Correlations Between Hematological and Serum Iron Status

**Figure 1:**
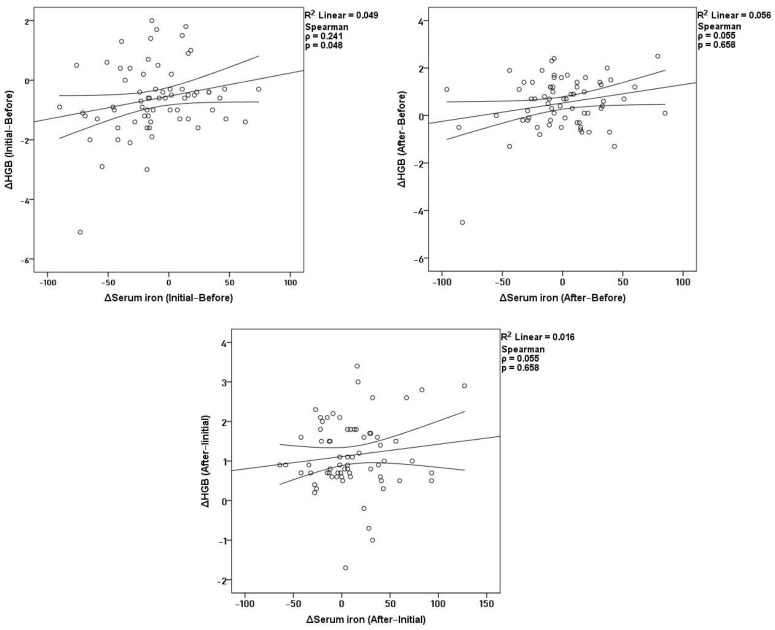
Correlations of Δ hemoglobin with Δ serum iron before treatment, after treatment, and across the full two-year.

Across the study period, the relationship between serum iron and hemoglobin showed a dynamic pattern that differed before and after dapagliflozin initiation (Figure 1). During the pre-treatment period (Δ Initial–Before), changes in serum iron demonstrated a weak but statistically significant positive correlation with changes in hemoglobin (*ρ* = 0.241, *p* = 0.048), indicating that declining hemoglobin levels were modestly accompanied by reductions in circulating iron before therapy. In contrast, during the post-treatment period (Δ After–Initial), no significant association was observed between changes in serum iron and changes in hemoglobin (*ρ* = 0.055, *p* = 0.658). When considering the net two-year interval (Δ After–Before), net changes in serum iron were likewise not significantly correlated with net changes in hemoglobin (*ρ* = 0.085, *p* = 0.492).

### 3.9. Correlations Between Hematological and Renal-Cardiac Parameters

#### 3.9.1. Pre-Treatment Correlations (Δ Initial–Before)

**Figure 2:**
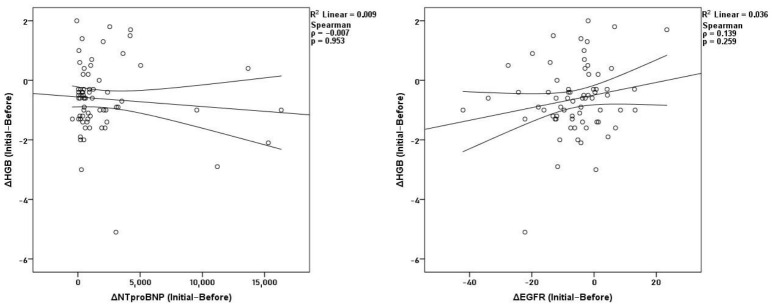
Correlations of Δ hemoglobin with ΔNT-proBNP and ΔeGFR before treatment.

In the year before starting dapagliflozin, hematological parameters showed a very consistent internal pattern. As expected, changes in hemoglobin strongly correlated with hematocrit (*ρ* = 0.781, *p* < 0.001) and red blood cell count (*ρ* = 0.715, *p* < 0.001), while hematocrit was also linked to RBC (*ρ* = 0.664, *p* < 0.001). No significant correlations were observed between hemoglobin and NT-proBNP (*ρ* = −0.007, *p* = 0.953) or eGFR (*ρ* = 0.139, *p* = 0.259) (Figure 2). Kidney and heart parameters were also not significantly related during this period. Overall, hematological, kidney, and heart changes mostly developed independently before treatment.

#### 3.9.2. Post-Treatment Correlations (Δ After–Initial)

**Figure 3:**
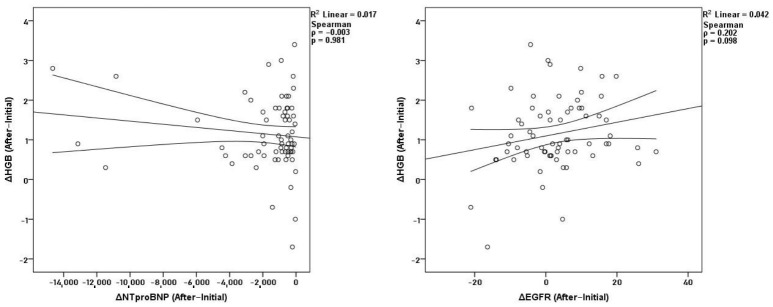
Correlations of Δ hemoglobin with ΔNT-proBNP and ΔeGFR after treatment.

After one year of dapagliflozin therapy, hematological correlations remained strong. Hemoglobin was closely associated with hematocrit (*ρ* = 0.875, *p* < 0.001) and RBC (*ρ* = 0.645, *p* < 0.001), and hematocrit correlated with RBC (*ρ* = 0.725, *p* < 0.001). Hemoglobin showed no significant correlation with NT-proBNP (*ρ* = −0.003, *p* = 0.981) or eGFR (*ρ* = 0.202, *p* = 0.098) (Figure 3). The only significant association across systems was an inverse correlation between ΔeGFR and ΔNT-proBNP (*ρ* = −0.327, *p* = 0.006). No additional renal-cardiac relationships were identified.

#### 3.9.3. Net Two-Year Correlations (Δ After–Before)

**Figure 4:**
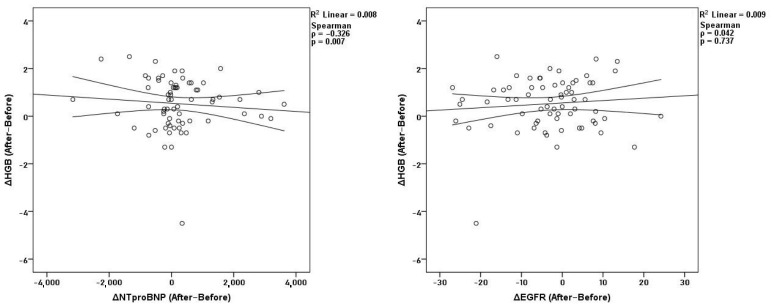
Correlations of Δ hemoglobin with ΔNT-proBNP and ΔeGFR before and after treatment.

Across the full two-year observation window, hematological parameters retained their internal consistency. Net changes in hemoglobin correlated strongly with hematocrit (*ρ* = 0.745, *p* < 0.001) and RBC (*ρ* = 0.634, *p* < 0.001), while hematocrit remained correlated with RBC (*ρ* = 0.636, *p* < 0.001). Additionally, hemoglobin showed an inverse correlation with NT-proBNP (*ρ* = −0.326, *p* = 0.007). Its relationship with eGFR remained non-significant (*ρ* = 0.042, *p* = 0.737) (Figure 4). No significant net correlations were observed between renal and cardiac parameters.

## 4. Discussion

In this self-controlled cohort of non-diabetic patients with heart failure, treatment with dapagliflozin was associated with a clear, clinically meaningful stimulation of erythropoiesis. All hematologic indices showed a consistent upward shift after one year of therapy, reversing the downward trajectory observed during the preceding untreated year. This directional change strongly suggests a treatment-related effect rather than background clinical fluctuation or regression toward the mean. Importantly, the increases in hemoglobin and hematocrit occurred independently of changes in renal or cardiac function. Importantly, this dissociation reflects a clinical-functional independence based on conventional measures of kidney and heart function and should not be interpreted as evidence of complete biological independence from renal mechanisms. Renal microvascular, cellular, or oxygen-sensing pathways may still contribute to erythropoiesis without being captured by global functional markers such as eGFR or NT-proBNP. No meaningful associations were observed between the hematologic response and variations in estimated glomerular filtration rate or NT-proBNP, despite modest improvements in both parameters over the same treatment period. In contrast, hematologic variables were tightly intercorrelated, indicating a coordinated erythropoietic response that evolved separately from cardiorenal physiology. Together, these findings indicate that the rise in erythropoiesis in this non-diabetic cohort is unlikely to be explained by decongestion, hemoconcentration, or improvements in heart or kidney function, but instead reflects a pharmacologic effect most plausibly attributable to SGLT2 inhibition.

To date, most mechanistic observations linking SGLT2 inhibition to erythropoiesis have emerged from studies in patients with type 2 diabetes (T2DM) [19,20,21]. In these populations, chronic hyperglycemia and elevated filtered glucose load augment proximal tubular sodium–glucose reabsorption, increase mitochondrial workload, and exacerbate intrarenal hypoxia, creating conditions in which interruption of SGLT2-mediated transport produces pronounced metabolic unloading [22]. As described by Packer and Vallon, reduced tubular energy demand restores cortical oxygenation, modulates hypoxia-inducible factor (HIF) signaling, and promotes erythropoietin (EPO) synthesis [15,16]. However, the relevance of these diabetes-specific mechanisms in non-diabetic individuals remains uncertain. In non-diabetic kidneys, tubular glucose reabsorption and the burden of glucotoxicity and mitochondrial dysfunction are substantially lower [23,24]. If reversal of hyperglycemia-driven tubular stress were the dominant driver of erythropoiesis, the magnitude of the hematologic response observed in our euglycemic cohort would be expected to be attenuated. Instead, our findings suggest a robust erythropoietic response despite the absence of hyperglycemic physiology, directly challenging this assumption and indicating that additional mechanisms must be operative.

Together, these observations indicate that the erythropoietic response to SGLT2 inhibition in non-diabetic heart failure patients cannot be explained solely through the classical diabetes-derived framework. The persistence of this hematologic effect in a euglycemic population suggests the involvement of additional pathways. Recent models propose renal [25], hepatic [14,20,26], and systemic mechanisms that may account for the rise in erythropoiesis [27,28]; however, most supporting evidence originates from diabetic populations [13,14,20,29,30]. In those settings, hyperglycemia profoundly shapes renal energetics, hepatic metabolism, iron handling, and inflammatory tone [31,32,33], making it difficult to separate direct drug effects from glycemic unloading [15,26,34]. Our non-diabetic cohort provides a clinical context in which these confounding metabolic factors are minimized, thereby strengthening the inference that at least part of the erythropoietic response reflects diabetes-independent pharmacologic effects.

Consistent with this concept, large outcome trials and meta-analyses demonstrate that the cardiorenal benefits of SGLT2 inhibitors are largely preserved irrespective of diabetes status [35,36,37]. Comparable reductions in CKD progression and acute kidney injury across glycemic strata suggest that sodium-dependent reductions in tubular workload, rather than glucose unloading per se, represent a dominant driver of renal metabolic relief in euglycemic physiology. Pooled analyses from the SGLT2 inhibitor Meta-Analysis Cardio-Renal Trialists’ Consortium and the NDPH Renal Studies Group confirm the absence of effect modification by diabetes status, with trials such as DAPA-CKD and EMPA-KIDNEY reporting similar benefits among non-diabetic participants [35,36,37]. In our cohort, renal function followed a biphasic trajectory, with a decline during the year preceding dapagliflozin initiation and subsequent stabilization or modest improvement after treatment, returning toward pre-decline values. This pattern mirrors the renal benefits observed in large outcome trials and is compatible with a reduction in intrarenal metabolic stress rather than a simple hemodynamic or glycemic effect. The dissociation between changes in eGFR and hematologic improvement further suggests that renal functional stabilization may coexist with, but does not directly mediate, the erythropoietic response. Nevertheless, mechanistic data in non-diabetic individuals remain sparse. Most insights into renal oxygenation, HIF signaling, and EPO dynamics continue to derive from diabetic models [38,39,40], and our study did not include biomarkers or imaging techniques capable of directly interrogating these pathways. Although clinical observations indicate that SGLT2 inhibitors increase hemoglobin and transiently stimulate EPO via HIF-2α activation in both diabetic and non-diabetic CKD [26,41], the precise cellular and oxygen-sensing mechanisms in non-diabetic kidneys remain incompletely defined. Thus, while clinical consistency supports a glycemia-independent effect, mechanistic confirmation is still largely inferential.

Improvement in renal microcirculatory dynamics may further contribute to erythropoiesis without requiring major changes in global renal function. SGLT2 inhibitors restore tubuloglomerular feedback, reduce afferent arteriolar vasodilation, and improve cortical oxygenation through increased sodium delivery to the macula densa [38,42,43], effects that are largely independent of glycemia [44]. Experimental studies demonstrate that these microvascular benefits persist in euglycemic models and are accompanied by reduced mitochondrial oxidative stress and selective HIF-2α modulation [26,28,39,45]. In non-diabetic heart failure patients, such changes may favor the preservation of EPO-producing interstitial fibroblasts [15,28,45,46]. However, direct clinical evidence linking renal microvascular changes to erythropoietin stimulation in non-diabetic patients is lacking, representing another important knowledge gap [26,35,37,47,48,49,50,51,52,53]. The dissociation observed in our cohort between erythropoietic changes and eGFR evolution is compatible with such microcirculatory or cellular effects occurring without measurable changes in global renal function. However, direct evaluation of renal microvascular dynamics was beyond the scope of this study.

Beyond renal mechanisms, emerging evidence suggests a potential hepatic contribution to SGLT2 inhibitor-induced erythropoiesis. In diabetic cohorts, SGLT2 blockade improves hepatic oxygenation, reduces inflammatory mediators, and enhances metabolic efficiency, changes that could plausibly stimulate hepatic EPO synthesis [15,54,55]. However, these hepatic effects frequently parallel improvements in hyperglycemia, steatosis, and lipotoxic stress, complicating mechanistic interpretation [15,54]. The persistence of a hematologic response in non-diabetic patients, where glucose levels were stable and hepatocellular glucotoxicity minimal, suggests that SGLT2 inhibitors may modulate hepatic oxygen sensing and inflammatory pathways independently of glycemic control [15,26]. Supporting this concept, hepcidin suppression and enhanced iron mobilization have been reported in both diabetic and non-diabetic populations [14,18,56]. No clinical studies have directly measured hepatic EPO production or HIF-2α activation in humans receiving SGLT2 inhibitors [15,26,39]. Thus, the hepatic contribution remains hypothetical and inferred from indirect observations. In our study, liver function was assessed longitudinally using serum transaminases. AST values remained stable across all three time points, while ALT showed a modest downward trend from the year preceding dapagliflozin initiation through one year of therapy, without reaching statistical significance. The absence of significant transaminase elevations suggests preserved hepatic integrity throughout the study period and argues against liver injury or overt hepatic stress as drivers of the hematologic response. Conversely, the lack of substantial improvement in liver enzymes indicates that major changes in hepatic metabolic function or steatosis are unlikely to explain the observed stimulation of erythropoiesis. These findings neither confirm nor exclude a hepatic contribution but suggest that, if present, such effects likely occur at the level of hepatic oxygen sensing or endocrine signaling rather than through measurable changes in conventional liver biochemistry.

A related hypothesis proposes that SGLT2 inhibitors directly stabilize the erythropoietin-producing phenotype of renal interstitial fibroblasts and prevent their transition into profibrotic myofibroblasts. In diabetic kidney disease, this maladaptive transition is driven by oxidative stress, TGF-β signaling, and inflammation, processes attenuated by SGLT2 inhibition in experimental models [57,58,59,60]. Beyond glucose lowering, SGLT2 inhibitors enhance HIF-2α signaling and preserve EPO expression in fibroblasts through mechanisms involving metabolic rewiring and reduced mitochondrial ROS [15,39,61,62]. Similar hematologic responses in non-diabetic populations suggest that these effects may extend beyond glycemic modulation. Nonetheless, direct lineage-tracing or human tissue studies confirming fibroblast stabilization are still unavailable [63,64,65].

Iron homeostasis represents another important pathway through which SGLT2 inhibitors may enhance erythropoiesis. In diabetic populations, reductions in hepcidin and increases in transferrin receptor expression are often linked to improved inflammation and metabolic stress [14,66]. Importantly, similar effects have been documented in non-diabetic heart failure cohorts, where SGLT2 inhibitors reduce hepcidin, increase iron mobilization, and activate the erythropoietin-erythroferrone-transferrin receptor axis [17,18,56]. The sustained erythropoietic response observed despite the absence of persistent increases in circulating EPO suggests that enhanced iron availability, rather than EPO alone, may be a critical driver of increased hemoglobin and hematocrit [18,56,67]. These effects are observed across heart failure phenotypes and appear independent of glycemic status [18,53]. Additionally, SGLT2 inhibitors have been shown to potentiate the erythropoietic response to intravenous iron in heart failure patients, suggesting a synergistic effect on iron metabolism and red cell production [68]. In our cohort, serum iron followed a biphasic trajectory, decreasing during the year preceding dapagliflozin initiation and subsequently increasing after one year of therapy, returning toward pre-decline values. This temporal pattern parallels the reversal observed for hematologic parameters and is compatible with improved iron availability following SGLT2 inhibition. However, changes in serum iron did not correlate with changes in hemoglobin after dapagliflozin initiation, suggesting that iron mobilization alone may not directly account for the magnitude of the erythropoietic response. It should be acknowledged that serum iron represents an incomplete surrogate of iron availability for erythropoiesis and does not capture the dynamic regulation of iron trafficking and utilization. The relationship between iron metabolism and erythropoiesis is highly complex and may be underestimated when assessed using isolated circulating iron measurements. Erythroferrone (ERFE) has emerged as a central mediator linking erythropoietic drive to iron homeostasis. Secreted by erythroblasts in response to erythropoietic stimulation, ERFE suppresses hepatic hepcidin expression, thereby increasing iron availability for effective erythropoiesis [69,70,71,72,73]. In heart failure and chronic kidney disease, inflammation promotes functional iron deficiency through hepcidin upregulation [56]. Recent data suggest that SGLT2 inhibitors may reactivate the erythropoietin–erythroferrone–hepcidin axis, enabling sustained erythropoiesis even in the absence of persistent elevations in circulating erythropoietin [15,18,56,67,74,75]. Although erythroferrone and hepcidin were not measured in the present study, the temporal pattern of hematologic improvement observed after dapagliflozin initiation is compatible with activation of this regulatory axis, which may reconcile the dissociation observed between serum iron dynamics and hemoglobin response.

Finally, the anti-inflammatory effects of SGLT2 inhibitors may act as a permissive mechanism for erythropoiesis. In diabetes, reductions in CRP, IL-6, and TNF-α often accompany improved glycemic control [76,77]. In non-diabetic heart failure, however, inflammation is driven predominantly by neurohormonal and hemodynamic factors, and SGLT2 inhibitors demonstrate anti-inflammatory activity independent of glucose lowering [45,78,79]. Because chronic inflammation suppresses erythropoiesis through hepcidin upregulation and iron restriction [80,81], attenuation of inflammatory signaling may enhance iron availability and bone marrow responsiveness. Supporting this interpretation, SGLT2 inhibitors reduce hepcidin and ferritin while increasing transferrin receptor expression in both diabetic and non-diabetic cohorts [14,18,56]. In our non-diabetic cohort, inflammatory status was systematically assessed using multiple circulating markers, including CRP, ESR, neutrophil-to-lymphocyte ratio (NLR), platelet-to-lymphocyte ratio (PLR), and the systemic immune–inflammation index (SII). Among these parameters, CRP demonstrated a modest but significant reduction after one year of dapagliflozin therapy, while ESR and composite inflammatory indices remained largely stable over time. However, the inflammatory markers assessed in this study reflect systemic, low-grade inflammation and are not specific for bone marrow inflammation or hepcidin-driven iron restriction. Therefore, while the observed reduction in CRP suggests attenuation of systemic inflammatory tone, these findings should be interpreted as permissive rather than mechanistically determinative for erythropoiesis.

Taken together, our findings suggest that dapagliflozin is associated with stimulation of erythropoietic activity in non-diabetic heart failure patients, in a manner that appears clinically independent of glycemia, cardiorenal improvement, or hemoconcentration. It should also be noted that the present cohort was not predominantly anemic at baseline. Therefore, while the observed increase in hemoglobin supports a pharmacologic erythropoietic effect, the direct contribution of hemoglobin elevation to cardiorenal improvement in this population may be limited. Whether SGLT2 inhibitor-induced erythropoiesis translates into clinically meaningful benefit in patients with established anemia remains an important question that warrants dedicated investigation. The consistency of the hematologic response suggests a multifactorial framework involving renal metabolic unloading, iron mobilization, microvascular and fibroblast preservation, and attenuation of systemic inflammation. While current evidence supports the plausibility of these pathways, our study clarifies the boundaries of current knowledge, as direct mechanistic confirmation in non-diabetic humans remains lacking. Our results reinforce emerging hypotheses while underscoring the need for dedicated mechanistic studies to define how SGLT2 inhibitors enhance erythropoiesis outside the context of diabetes.

## 5. Strengths and Limitations of the Study

This study has several notable strengths that enhance both the robustness and the clinical relevance of the findings. First, the self-controlled, within-patient design allowed each participant to serve as their own comparator across three predefined time points, thereby minimizing interindividual variability and reducing confounding related to baseline demographic, clinical, and therapeutic differences. This approach is particularly valuable in real-world heart failure populations, where clinical heterogeneity is substantial and randomized designs are often difficult to implement. Importantly, dapagliflozin represented the only therapeutic modification during the observation period, with no other changes in guideline-directed medical therapy, minimizing confounding related to background treatment adjustments and strengthening the temporal association between treatment initiation and the observed hematologic response. Second, the exclusive inclusion of non-diabetic patients represents a major strength and directly addresses an important gap in the existing literature. Most prior studies investigating SGLT2 inhibitor-associated erythropoiesis have been conducted in diabetic or mixed populations, making it difficult to disentangle glycemia-dependent from glycemia-independent mechanisms. By focusing on a strictly non-diabetic cohort, our study provides clinically relevant evidence that the erythropoietic response to dapagliflozin extends beyond the context of hyperglycemic physiology. Third, the longitudinal assessment spanning two consecutive years, one preceding and one following dapagliflozin initiation, allowed characterization of temporal trajectories rather than isolated cross-sectional comparisons. The observation that hematologic parameters were declining before treatment initiation and subsequently reversed after dapagliflozin introduction strengthens the inference of a treatment-associated effect and reduces the likelihood that the findings reflect spontaneous fluctuation or regression toward the mean. Fourth, the comprehensive and parallel evaluation of multiple biological domains represents a key strength of this study. In addition to standard hematologic, renal, and cardiac parameters, we longitudinally assessed liver enzymes, systemic inflammatory markers (CRP, ESR, NLR, PLR, SII), and serum iron status. This multidimensional approach enabled an integrated analysis of inter-system relationships and allowed us to contextualize the erythropoietic response within broader metabolic, inflammatory, and iron-related frameworks. The demonstration that erythropoietic changes occurred independently of renal function, cardiac status, systemic inflammation, liver enzyme variation, and serum iron correlations provides important mechanistic insight and supports the concept of a direct pharmacologic effect rather than a secondary consequence of decongestion, cardiorenal recovery, or isolated iron mobilization. Finally, the study reflects a real-world heart failure population treated with contemporary guideline-directed medical therapy, enhancing external validity and translational relevance. The consistency of the erythropoietic response across this clinically complex cohort, despite heterogeneity in comorbidities and baseline risk, underscores the potential generalizability of the findings and their relevance to routine clinical practice.

Despite these strengths, several limitations should be acknowledged, particularly with respect to mechanistic interpretation. First, the observational, self-controlled design precludes definitive causal inference, and mechanistic attribution remains indirect. Second, the study did not include direct assessments of renal oxygenation, tubular workload, or hypoxia-inducible factor signaling, limiting confirmation of proposed renal metabolic mechanisms in non-diabetic patients. Third, hepatic involvement could not be directly evaluated, as no hepatic biomarkers beyond routine transaminases, imaging data, or source-specific erythropoietin measurements were available. Similarly, the absence of tissue-level data or lineage-tracing biomarkers precluded direct validation of fibroblast-related mechanisms. Finally, although serum iron was assessed longitudinally, the lack of serial measurements of key iron-regulatory mediators, including hepcidin, ferritin, transferrin saturation, and erythroferrone, limits direct confirmation of iron-mediated pathways. Moreover, more specific contemporary inflammatory biomarkers in heart failure, such as interleukin-6 (IL-6) or high-sensitivity C-reactive protein (hsCRP), were not available, which may further limit refined characterization of inflammation-related and hepcidin-dependent mechanisms. In addition, although the sample size was appropriate for a longitudinal observational study, it may have limited statistical power to detect weaker associations, particularly in correlation analyses, thereby raising the possibility of a type II error and limiting the generalizability of negative findings. Collectively, these limitations underscore that the mechanistic interpretations proposed herein remain inferential and highlight the need for dedicated prospective studies incorporating renal, hepatic, inflammatory, and iron-regulatory endpoints in non-diabetic populations.

## 6. Conclusions

In this cohort of non-diabetic patients with heart failure, dapagliflozin induced a significant and sustained increase in hemoglobin, hematocrit, and red blood cell count, independent of changes in renal function or cardiac status. These findings provide real-world evidence that stimulation of erythropoiesis represents a diabetes-independent pharmacologic effect of SGLT2 inhibition rather than a secondary consequence of decongestion or cardiorenal recovery.

The dissociation between hematologic changes and renal, cardiac, inflammatory, hepatic, and iron-related parameters supports a multifactorial mechanism that likely involves renal metabolic unloading, modulation of oxygen-sensing pathways, permissive effects on iron handling, and attenuation of systemic stress signals, rather than a single dominant pathway. While these mechanisms are biologically plausible and consistent with emerging experimental and clinical data, direct mechanistic confirmation in non-diabetic humans remains lacking.

Future prospective and mechanistic studies incorporating renal and hepatic oxygenation, erythropoietin dynamics, iron-regulatory pathways, and inflammatory signaling are needed to clarify how SGLT2 inhibitors enhance erythropoiesis outside the context of diabetes and to define the clinical implications of this response. Elucidating these pathways will be essential for a more complete understanding of the pleiotropic biological effects of SGLT2 inhibition beyond glucose regulation.

## Figures and Tables

**Table 1:** Baseline characteristics.

Total Patients—No.	68
Age—years (SD)	70.69 (10.92)
Female sex—no. (%)	40 (58.8%)
Urban environment—no. (%)	30 (44.1%)
BMI—kg/m2 (SD)	29.12 (6.22)
Hypertension—no. (%)	
Without—no. (%)	7 (10.3%)
Grade 1—no. (%)	4 (5.9%)
Grade 2—no. (%)	43 (63.2%)
Grade 3—no. (%)	14 (20.6%)
Dyslipidemia—no. (%)	20 (29.4%)
NAFLD—no. (%)	25 (36.8%)
Chronic kidney disease—no. (%)	
Without—no. (%)	41 (60.3%)
KDIGO G2	6 (8.8%)
KDIGO G3a	5 (7.4%)
KDIGO G3b	14 (20.6%)
KDIGO G4	2 (2.9%)
Atrial fibrillation—no. (%)	36 (52.9%)
Treatment—no. (%)	
ACEi/ARA	58 (85.29%)
Sacubitril + Valsartan	17 (25%)
Beta Blocker	62 (91.17%)
Loop diuretic	42 (61.76%)
MRA	48 (70.58%)
Statin	49 (72.05%)

Abbreviations: BMI—body mass index, NAFLD—non-alcoholic fatty liver disease, ACEi—angiotensin convertase enzyme inhibitor, ARA—angiotensin receptor antagonist, MRA—mineralocorticoid receptor antagonist.

**Table 2:** Evolution of cardiovascular parameters before and after one year of dapagliflozin treatment.

Parameters	1 Year Before	Dapagliflozin Initiation	1 Year After
NYHA Classification	2 (1–2)	3 (2–3) **^a^**	2 (2–2) **^b^**
LVEF	52% (42.25–55%)	42% (36–49.75%) **^a^**	50.5% (45–55.75%) **^b^**
NT-proBNP (pg/mL)	451.55 (193.25–1368.90)	1761.19 (714.60–3181.20) **^a^**	694.80 (278.98–1396.91) **^b^**

Values are expressed by the median and the 25th, 75th percentiles. **^a^** *p* < 0.001 compared to 1 year before values; **^b^** *p* < 0.001 compared to initial values.

**Table 3:** Evolution of renal parameters before and after one year of dapagliflozin treatment.

Parameters	1 Year Before	Dapagliflozin Initiation	1 Year After
Serum urea (mg/dL)	42.40 (31.30–52.00)	41.25 (30.72–58.02)	38.60 (29.82–45.27) **^a,b^**
Serum creatinine (mg/dL)	0.83 (0.75–1.07)	0.92 (0.77–1.19) **^a^**	0.88 (0.79–1.08) **^b^**
eGFR (mL/min/1.73 m^2^)	74.65 (58.80–89.45)	68.05 (52.82–85.22) **^a^**	75.40 (54.92–85.17) **^a,b^**

Values are expressed by the median and the 25th, 75th percentiles. **^a^** *p* < 0.05 compared to 1 year before values; **^b^** *p* < 0.05 compared to initial values.

**Table 4:** Evolution of liver parameters before and after one year of dapagliflozin treatment.

Parameters	1 Year Before	Dapagliflozin Initiation	1 Year After
AST	21.50 (19.00–26.75)	21.00 (17.00–26.75)	22.00 (17.25–27.00)
ALT	21.00 (14.00–25.00)	18.50 (14.00–23.75)	17.00 (14.00–23.75)

Values are expressed by the median and the 25th, 75th percentiles.

**Table 5:** Evolution of inflammatory parameters before and after one year of dapagliflozin treatment.

Parameters	1 Year Before	Dapagliflozin Initiation	1 Year After
CRP	2.88 (1.48–4.00)	3.21 (1.48–5.46)	2.38 (0.96–4.45) **^b^**
ESR (mm/h)	15.50 (6.25–23.75)	13.50 (8.00–21.75)	14.00 (7.00–21.00)
NLR	2.39 (1.85–3.16)	2.43 (1.95–3.26)	2.47 (1.66–3.17)
PLR	110.14 (85.85–146.95)	119.12 (93.77–151.81)	122.18 (91.21–159.84)
SII	464.78 (336.20–769.85)	531.71 (393.04–644.54)	545.12 (332.04–746.67)

Values are expressed by the median and the 25th, 75th percentiles. **^b^** *p* < 0.05 compared to initial values.

**Table 6:** Evolution of hematological parameters before and after one year of dapagliflozin treatment.

Parameters	1 Year Before	Dapagliflozin Initiation	1 Year After
RBC (10^6^/µL)	4.71 (4.25–4.90)	4.43 (3.97–4.77) **^a^**	4.75 (4.37–5.13) **^a,b^**
Hemoglobin (g/dL)	13.75 (12.72–14.87)	13 (12.10–14.07) **^a^**	14.3 (13.32–15.30) **^a,b^**
Hematocrit	42.00% (38.52–44.55%)	40.40% (36.17–42.72%) **^c^**	43.65% (40.52–46.20%) **^a,b^**
MCV (fL)	91.15 (84.90–95.05)	92.10 (87.60–95.50)	92.20 (88.17–94.90)
MCH (pg)	30.20 (28.12–31.77)	30.60 (28.92–31.47)	30.30 (29.02–31.27)
MCHC (g/dL)	33.10 (32.32–34.45)	33.10 (32.20–33.70) **^d^**	32.75 (32.30–33.37) **^d,e^**

Values are expressed by the median and the 25th, 75th percentiles. **^a^** *p* < 0.001 compared to 1 year before values; **^b^** *p* < 0.001 compared to initial values; **^c^** *p* = 0.005 compared to initial values; **^d^** *p* < 0.05 compared to initial values; **^e^** *p* < 0.05 compared to 1 year before values.

**Table 7:** Evolution of serum iron before and after one year of dapagliflozin treatment.

Parameters	1 Year Before	Dapagliflozin Initiation	1 Year After
Serum iron (mg/dL)	84.50 (70.25–110.00)	75.50 (53.25–98.75) **^a^**	84.00 (64.50–103.75) **^b^**

Values are expressed by the median and the 25th, 75th percentiles. **^a^** *p* < 0.05 compared to 1 year before values; **^b^** *p* < 0.05 compared to initial values.

## Data Availability

The original contributions presented in this study are included in the article. Further inquiries can be directed to the corresponding author.
